# Flow in human-robot collaboration—multimodal analysis and perceived challenge detection in industrial scenarios

**DOI:** 10.3389/frobt.2024.1393795

**Published:** 2024-05-30

**Authors:** Pooja Prajod, Matteo Lavit Nicora, Marta Mondellini, Matteo Meregalli Falerni, Rocco Vertechy, Matteo Malosio, Elisabeth André

**Affiliations:** ^1^ Human-Centered Artificial Intelligence, University of Augsburg, Augsburg, Germany; ^2^ Institute of Intelligent Industrial Technologies and Systems for Advanced Manufacturing, National Research Council of Italy, Lecco, Italy; ^3^ Industrial Engineering Department, University of Bologna, Bologna, Italy; ^4^ Psychology Department, Catholic University of the Sacred Heart, Milan, Italy

**Keywords:** human-robot interaction, flow, heart rate variability, emotion estimation, collaboration, industry 5.0, task load, machine learning

## Abstract

**Introduction:** Flow state, the optimal experience resulting from the equilibrium between perceived challenge and skill level, has been extensively studied in various domains. However, its occurrence in industrial settings has remained relatively unexplored. Notably, the literature predominantly focuses on Flow within mentally demanding tasks, which differ significantly from industrial tasks. Consequently, our understanding of emotional and physiological responses to varying challenge levels, specifically in the context of industry-like tasks, remains limited.

**Methods:** To bridge this gap, we investigate how facial emotion estimation (valence, arousal) and Heart Rate Variability (HRV) features vary with the perceived challenge levels during industrial assembly tasks. Our study involves an assembly scenario that simulates an industrial human-robot collaboration task with three distinct challenge levels. As part of our study, we collected video, electrocardiogram (ECG), and NASA-TLX questionnaire data from 37 participants.

**Results:** Our results demonstrate a significant difference in mean arousal and heart rate between the low-challenge (Boredom) condition and the other conditions. We also found a noticeable trend-level difference in mean heart rate between the adaptive (Flow) and high-challenge (Anxiety) conditions. Similar differences were also observed in a few other temporal HRV features like Mean NN and Triangular index. Considering the characteristics of typical industrial assembly tasks, we aim to facilitate Flow by detecting and balancing the perceived challenge levels. Leveraging our analysis results, we developed an HRV-based machine learning model for discerning perceived challenge levels, distinguishing between low and higher-challenge conditions.

**Discussion:** This work deepens our understanding of emotional and physiological responses to perceived challenge levels in industrial contexts and provides valuable insights for the design of adaptive work environments.

## 1 Introduction

Flow or Effortless attention is often described as a state of optimal experience. It is characterized by high levels of engagement, a sense of control, and complete immersion in an activity (Csikszentmihalyi, 2000; Nakamura and Csikszentmihalyi, 2002; Csikszentmihalhi, 2020). This state emerges when the challenges presented by the task match the individual’s skills and abilities. Flow is associated with a feeling of intrinsic motivation, enjoyment, and focused attention, leading to improved performance and a positive experience.

While extensive research (Nah et al., 2014; Stamatelopoulou et al., 2018; dos Santos et al., 2018; Pearce, 2005) has been conducted on the concept of Flow across various domains, such as sports, education, and gaming, its application in industrial settings remains relatively unexplored. Moreover, the tasks studied in the literature on Flow experience are mentally demanding, which is not typical in an industrial task. Considering the significance of Flow in optimizing performance and wellbeing at work (Csikszentmihalyi and LeFevre, 1989; Csikszentmihalhi, 2020), it is imperative to bridge this research gap and explore the Flow experience in industrial environment (Fullagar et al., 2018; Beyrodt et al., 2023).

To address this gap, we designed an assembly task within a laboratory work cell, closely resembling an industrial setting. This task involved collaboration between a participant and a collaborative robot (cobot) to assemble gearboxes. By adjusting the production rate of the cobot, we created three distinct levels of challenge that correspond to the three commonly studied states in Flow research (Boredom, Flow, and Anxiety). This enables us to analyze the emotional and physiological responses to different challenge levels during an industry-like human-robot collaboration (HRC) task.

The assembly tasks in industrial settings typically involve repetitive and fixed procedures. As a result, workers gradually acquire the necessary skills to proficiently perform the task, leading to minimal variations in their individual skill levels over time. In such cases, the primary factor that influences the experience of Flow becomes the perceived level of challenge presented by the tasks. This unique aspect of industrial tasks has led us to specifically investigate how different perceived challenge levels evoke distinct responses. Recognizing that Flow emerges when there is a balance between perceived challenge and skill, our goal is to adapt the task by adjusting the challenge level to facilitate flow among cobot workers.

Recent studies (Lee, 2020; Rissler et al., 2020; Di Lascio et al., 2021) have explored the automatic detection of flow at work through physiological signals. They detect either the presence of Flow (Flow vs. No Flow) or classify the intensity of Flow (low vs. high). However, considering the specific characteristics of industrial assembly tasks, we have taken a different approach—detecting the level of perceived challenge. This approach aligns logically with our objective of adapting the task’s challenge level. Moreover, manipulating the perceived challenge of a task is typically easier than manipulating the flow experience itself. This is partly because while achieving a balance between challenge and skill is necessary for flow, it is not adequate by itself.

In summary, our contributions involve investigating facial emotion estimation (valence and arousal) and heart rate variability as indicators of perceived challenge levels within the context of industrial assembly tasks. Through our analysis, we have trained a model to predict perceived challenge levels. Our findings hold the potential to inform the design of adaptive work environments, dynamically adjusting challenge levels based on real-time feedback, thus fostering optimal worker experiences.

## 2 Background and related work

### 2.1 Concept of flow

**FIGURE 1:**
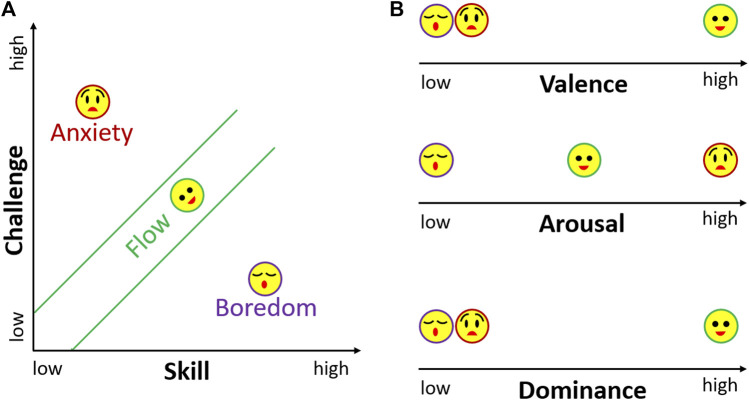
**(A)** An illustration of Boredom, Flow, and Anxiety states in terms of Challenge and Skill, commonly known as Csikszentmihalyi’s three-channel Flow model. **(B)** A mapping of Boredom, Flow, and Anxiety on emotional dimensions of Valence, Arousal, and Dominance based on the literature.

Flow is a state of optimal experience that is conducive to improving performance and positive experiences. Studies have identified many characteristics of Flow including intense focus, immersion, and a sense of control (Nakamura and Csikszentmihalyi, 2002; Csikszentmihalhi, 2020; Lee, 2020). One of the necessary conditions for Flow is the balance between Challenge and Skill, i.e., the individual’s skill level matches the level of challenge of the task. As illustrated in Figure 1, the imbalance between challenge and skill can lead to negative experiences of Boredom and Anxiety. On one hand, when the task challenge is higher than the skill level of the individual, it leads to Anxiety. On the other hand, when the challenge is much lower than the individual’s skill level, it leads to feelings of Boredom. Although studies have identified other experiences in the challenge-skill model, we consider the simple three-channel Flow model (Csikszentmihalyi, 2000; Pearce, 2005) in this study.

Some studies have confirmed the correlations between physiological arousal and the states in the challenge-skill model (Peifer et al., 2014; Tozman et al., 2015). Specifically, Peifer et al. proposed a model where arousal increases from Boredom to Anxiety (see Figure 1). In other words, Boredom is a low arousal state, Flow can be viewed as a moderate arousal state and Anxiety is a high arousal state. Some studies (Nacke and Lindley, 2008; Mauri et al., 2011) also showed Flow to be a positive valence, high arousal state. Furthermore, studies such as Gilroy et al. (2009) and Beyrodt et al. (2023) characterize Flow as a positive dominance and positive valence emotional state, distinguishing it from Boredom and Anxiety, which are associated with negative dominance and negative valence. This motivated our exploration of emotion estimation values for distinguishing Boredom, Anxiety, and Flow states.

### 2.2 Flow and heart rate variability

Many studies have investigated the relationship between Flow and various physiological signals like cardiovascular function, Electromyography (EMG), respiration, etc. (Kivikangas et al., 2006; De Manzano et al., 2010; Peifer, 2012; Gaggioli et al., 2013). Among the physiological signals, the most promising and well-researched signal is Heart Rate Variability (HRV). HRV is computed from the variation in time intervals (typically measured in milliseconds) between successive heartbeats. HRV can be computed from Electrocardiogram (ECG) signals obtained via chest-worn devices or from Photoplethysmography (PPG) or Blood Volume Pulse (BVP) measurements captured using hand-worn devices.

EDA (Electrodermal activity) is another physiological signal typically employed in real-time flow detection models. However, in the context of Flow detection, HRV has a higher distinguishing capability than EDA (Knierim et al., 2018). Moreover, as seen in the next section (Section 2.3), incorporating EDA does not necessarily improve the Flow detection performance. Hence, in this work, we primarily focus on the relationship between Flow and HRV.

The previous research mentioned below has linked Flow to the activation of the autonomic nervous system (ANS). Both sympathetic activation (resulting in arousal) and parasympathetic activation (resulting in relaxation) of the ANS have been associated with the various states of the Flow model (Knierim et al., 2018). Specifically, sympathetic activation is associated with increased heart rate (HR), while parasympathetic activation is correlated with decreased heart rate (Pham et al., 2021). HRV features are known indicators of both types of activation, making them well-suited for analyzing experiences within the Flow model. Flow state is often associated with an increase in HR and a reduction in inter-beat intervals or mean HRV (Tian et al., 2017). Although many studies have investigated the low-frequency (LF) and high-frequency (HF) components of HRV, there is no clear consensus on how these features vary with flow.

De Manzano et al. (2010) were one of the first to investigate physiological responses in Flow states. They studied HRV, facial EMG, and respiration of professional pianists in their experiment. They investigated mean and power spectrum (LF/HF, total power) features of HRV and found significant correlations between all three measures and Flow. Building on the work by De Manzano et al., Jha et al. (2022) studied pianists’ HRV (low and high frequencies) derived from ECG collected before, during, and after a performance. They also found a trend-level positive correlation between LF/HF and Flow.

Keller et al. (2011) studied how HRV features varied between Boredom, Anxiety, and Flow conditions of a knowledge task (computerized quiz game). They experimentally demonstrated the relationship between mean HRV and mental task load. Boredom led to highest mean HRV, and the other two conditions showed a decrease in HRV. They also collected cortisol measurements to determine whether the decreased HRV in the Flow condition was due to engagement or mental strain. They found that the cortisol measurements were similar to the Anxiety condition, suggesting a stress response.

Building on the observations of Keller et al., Peifer et al. (2014) demonstrated the relationship between Flow and physiological arousal using HRV features. They utilized a game which the participants played after experiencing a stressful situation. They found that the flow experience under stress involves both moderate arousal (low-frequency) and increased relaxation (high-frequency) in HRV, indicating both sympathetic and parasympathetic activations in Flow state.

Expanding on previous research, several studies employed games with varying challenge levels (Boredom, Anxiety, and Flow) to investigate participants’ physiological responses. The findings corresponding to their HRV analyses are summarized here. While Harmat et al. (2015) suggested a link between Flow and reduced LF, their findings lacked statistical significance. In contrast, other studies such as Bian et al. (2016), Tian et al. (2017), and de Sampaio Barros et al. (2018) reported a linear relationship between task difficulty and heart rate (lowest during Boredom, highest during Anxiety) and mean HRV (highest during Boredom, lowest during Anxiety). Additionally, Bian et al. observed an inverted U-shaped relationship between Flow and both LF and HF components of HRV.

Similar to gaming scenarios, Tozman et al. (2015) investigated how the low- and high-frequency components of HRV varied during Boredom, Anxiety, and Flow conditions. They utilized a virtual driving simulator to induce the three conditions. They found that both LF and HF components decreased with increasing task difficulty.

Moving away from laboratory situations, Gaggioli et al. (2013) investigated Flow-experience during daily activities of university students. They found correlations between Flow and certain HRV features (heart rate and LF/HF ratio). However, they did not analyze the type of activities or the challenge (mental, physical, etc.).

In their study, Knierim et al. (2019) explored the experience of flow in arithmetic (boredom, flow, overload) and scientific writing tasks. They analyzed RMSSD (lower values imply higher stress) and HF features of HRV. Results showed higher RMSSD in the flow condition compared to overload in arithmetic tasks, indicating lower stress. Moreover, they observed a trend-level decrease in HF with increasing challenge, consistent with previous findings. However, these features remained consistent throughout the writing task, but were lower compared to arithmetic tasks, suggesting a higher arousal state. This study underscores the variability in flow experience even within similar tasks.

The focus of most of these works is the relationship between Flow and HRV features involving mentally demanding tasks. However, assembly line tasks in an industrial setting seldom involve a high mental load. Thus, it is yet to be investigated whether similar HRV variations can be observed during varying challenges of assembly tasks.

### 2.3 Detecting flow at work

Some studies have proposed automatic Flow detection models using physiological signals in the context of games (Chanel et al., 2008; Chatterjee et al., 2016; Maier et al., 2019). They typically induce varying difficulty/challenge levels in the game and classify the corresponding physiological data. Recently, a few studies described below have proposed Flow detection models for activities that occur in the workplace.

Müller and Fritz (2015) proposed prediction of progress (Stuck vs. in Flow) during software programming tasks. They used various physiological signals including HRV (from BVP), pupil features, and EDA, and obtained an accuracy of 67.7%. Lee (2020) studied the classifications of Flow using signals similar to Müller and Fritz in a lab study involving researchers and graduate students doing knowledge tasks (editing spreadsheets, reading and summarizing, answering patent questions, etc.).

Rissler et al. (2020) proposed machine learning models to detect low-flow vs. high-flow using ECG-based HRV features during work scenarios. They conducted two experiments - one in the lab and the other in-the-wild. For the lab experiment, they recreated an invoice-matching task where participants had to match invoices with corresponding payments. They induced challenges by varying levels of arithmetic calculations. For the in-the-wild study, they recruited software developers doing their regular work activities. They achieved an accuracy of 68.5% in the lab setting and 70.6% for the in-the-wild study.

Inspired by Rissler et al., Di Lascio et al. (2021) investigated the use of physiological signals (BVP, EDA) along with context information to predict low and high Flow instances. They studied the daily activities of university employees (professors, researchers, PhDs). Considering the individual modalities, they obtained the best accuracy of 67.46% using HRV features. Their best model (accuracy of 70.93%) was a fusion of raw BVP, EDA, and context information. Notably, their accuracy is on par with Rissler et al., who used only HRV features to detect Flow.

All the above-mentioned studies focus on mentally demanding tasks and often involve specific groups like researchers or software developers. Neither the tasks nor the participant groups are similar to the industrial scenario that we target in our study. Additionally, we note that the models proposed attempt to detect Flow (Flow vs. No-Flow, Low-Flow vs. High-Flow), plausibly because of low control over the challenge levels in the study scenarios. However, the scenario we consider is more similar to the games mentioned previously, where the cobot behavior and the corresponding challenge levels can be better controlled. Hence, we adopt an approach of classifying perceived challenge levels rather than detecting Flow.

### 2.4 Research questions

Based on the gaps identified in the literature, we formulate two research questions. The first question addresses the need for analyzing the emotional and physiological responses to varying levels of challenge during an industry-like human-robot collaboration scenario. It is not clear whether responses would be similar to the tasks where the challenges depend mainly on mental load.

**RQ 1a.** Are there differences in the emotional responses (valence and arousal) of participants during different levels of challenge?

**RQ 1b.** Are there differences in the physiological responses (heart rate variability features) of participants during different levels of challenge?

The second question addresses the feasibility of developing a model to predict the perceived challenge level based on the emotional and physiological data. Such a model could be employed to adapt the cobot behavior to provide an optimal challenge that matches the skill level of the operator and thus supports the occurrence of Flow state.

**RQ 2.** Can we train a model with accuracy comparable to the state-of-the-art Flow detection models to predict the perceived challenge level using the emotional and physiological data?

## 3 Materials and methods

### 3.1 Experimental setup

**FIGURE 2:**
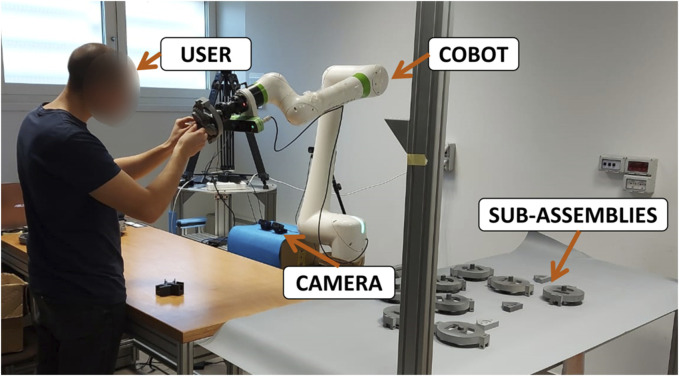
An overview of the experimental setup where the participant is assembling one product in collaboration with a cobot. The frontal webcam is used to record a video of the participant’s face during the interaction. The participant is also wearing a chest band (not visible) under the t-shirt that records ECG.

**FIGURE 3:**
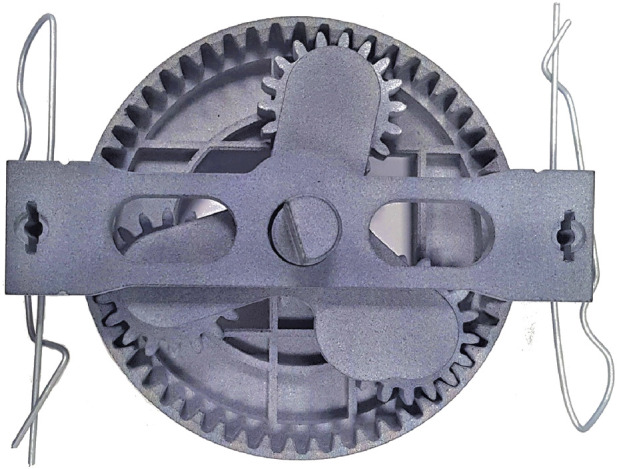
A picture of one finished planetary gearbox assembled collaboratively by both the participant and the cobot.

The study is carried out in a lab-based environment set up to emulate an industrial collaborative robotic workcell. Figure 2 shows a participant working in direct contact with a Fanuc CRX-10iA/L cobot (an industrial robotic arm) mounted in the corner of an L-shaped table arrangement. The table in the front is used by the participant for his/her part of the assembly, while the table on the side is equipped with a matrix of pre-assembled components (sub-assemblies) ready to be picked up by the cobot. The product to be collaboratively assembled is the planetary gearbox (Redaelli et al., 2021) represented in Figure 3. Half of the components are put together manually by the participant, while the other half is pre-assembled on the cobot’s table as if produced by an additional part of the production line that has not been reproduced in this laboratory setup. We decided to undertake this approach in order to have more flexibility in the production rate of the cobot for the tested experimental conditions, as explained in detail in Section 3.2.

During each production cycle, the cobot’s task is to bring a pre-assembled part to the participant and hold it in a convenient position for the final joint activity of the production cycle (gears meshing), as represented in Figure 2. The cobot is equipped with a detection camera (Pickit3D camera) and a gripper (Robotiq Hand-e parallel gripper) mounted on its wrist enabling it to pick up one of the pre-assembled sub-assemblies and bring it to the participant. There were no locating errors because the matrix positions were pre-determined and known to the cobot. During the joint activity, the cobot stayed in the joint meshing position until the participant pressed a foot switch to trigger the release of meshed sub-assemblies. The cobot performed a scanning motion over the sub-assembly matrix during idle periods (i.e., when it was not performing actions for the joint meshing).

We collected three types of data to evaluate how the participants responded to the experimental conditions—face video, ECG data, and NASA-TLX (Hart and Staveland, 1988) questionnaire. A Logitech C920 Pro HD webcam is placed in front of the participant (around 1.5 m away) and used to record their face videos in 1920 × 1080 at 25 frames per second. The participants wore a Polar H10 chest band that captured their ECG signals at 130 Hz. The chest band was paired with an Android phone to receive and store the raw signals.

### 3.2 Experimental conditions

While previous works (Kulić and Croft, 2007; Arai et al., 2010; Koppenborg et al., 2017; Kühnlenz et al., 2018; Gervasi et al., 2022; Zakeri et al., 2023) in HRC investigated a robot’s movement speed and proximity as factors that induce stress and anxiety, these factors have not been shown to induce boredom or flow. Based on our previous exploratory study (Mondellini et al., 2023), we identified production rate as a factor influencing boredom and flow, in addition to anxiety. We observed three distinct scenarios based on the production rate of the participant and the cobot—participant waiting for the cobot, cobot waiting for the participant, and synchronized assembly. We translated these scenarios into three distinct challenge levels of the assembly task to elicit the states in the simplified Flow model. The cobot behavior was modified to achieve these three challenge levels, as described below.

1. **Slow condition**: The cobot performs a scanning motion over all the sub-assemblies using the camera on its wrist before picking one of them up and bringing it to the user. Overall, it takes around 55 s from the start of each production cycle for the cobot to get to the participant for the joint activity.
2. **Fast condition**: The cobot does not perform any scanning motion, it moves straight to the next sub-assembly to pick up and bring it to the participant. Overall, it takes around 15 s from the start of each production cycle for the robot to get to the participant for the joint activity.
3. **Adaptive condition**: The cobot performs the previously mentioned scanning motion until the researcher, acting as a Wizard of Oz, triggers it to bring one sub-assembly to the participant. There is no fixed timing for the cobot in this case, i.e., the wizard triggers the cobot whenever the participant is close to finishing his/her part of the assembly.

The Slow condition represents a low level of challenge for the participants since they have plenty of time to finish their part of the assembly before the cobot comes for the joint activity. This leads to the participant waiting for the cobot and plausible experience of Boredom. The Fast condition is expected to be perceived by the participants as a high level of challenge since they do not have enough time to assemble before the arrival of the cobot. This leads to the cobot waiting for the participant and could elicit Anxiety in the participants. The Adaptive condition is designed to be the optimal level of challenge since the production rate of the cobot is tuned according to the participant’s performance. The experimental conditions and the associated emotional states were validated in another study (Beyrodt et al., 2023).

### 3.3 Experimental protocol

The experiment started with a preparation phase where the participants were introduced to the setup and the task. They were briefed about the data that would be collected and subsequently were administered the informed consent form. They were also informed that they could withdraw their participation at any point during the experiment. After signing the consent form, we collected their demographic information. They were also requested to wear the chest band for collecting ECG data. In this phase, the participants were encouraged to practice the assembly task until they were familiar with the task. A researcher was present in the room throughout the experiment to fix any technical errors that may occur during the sessions. He was also the Wizard in the Adaptive condition. He was fluent in both Italian and English. Depending on the participant’s preference, the instructions and questionnaire were provided in Italian or English. He avoided any superfluous interaction with the participants and the participants were requested to defer any comments about the experimental sessions till the end of the experiment.

**FIGURE 4:**
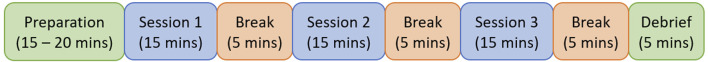
An overview of the experimental protocol consisting of three conditions (Slow, Fast, Adaptive). To mitigate any ordering effect, the condition sequences were counterbalanced among participants.

As illustrated in Figure 4, we follow a within-subjects design. Every participant is administered all three experimental conditions with 5 min of break between consecutive sessions. Each condition lasts 15 min during which the participant keeps assembling gearboxes one after the other. The order in which the three conditions are administered is chosen randomly, in order to average out any side effect that may be caused by the sequence. During the break, the participants fill out the NASA-TLX questionnaire about the task load and experience pertaining to the completed session. After the three sessions, the participants are debriefed about the experiment.

### 3.4 Participants and ethics

A total of 37 adult volunteers (8 females and 29 males) aged 18–48 years (mean = 29.03, SD = 7.08) were recruited for the study. The participants were students and staff from the National Research Council—Lecco campus and were mostly Italians (Italians—33, non-Europeans—4). All the participants were recruited through word-of-mouth and advertising in public areas. Note that none of the participants had prior experience working with an industrial cobot.

The study has been conducted according to the guidelines of the Declaration of Helsinki and approved by the institute’s ethics committee (protocol 0085720/2022). All the participants were briefed about the study and the details of data treatment before signing an informed consent form.

### 3.5 NASA-TLX questionnaire

The NASA-TLX questionnaire comprises six sub-scales that represent the factors relating to workload: Mental Demand, Physical Demand, Temporal Demand, Frustration, Effort, and Performance. Each sub-scale is rated from 1 (very low) to 20 (very high). We chose to use this questionnaire as it indicates what type of task load is experienced by the participants. As discussed in Section 2, the studies in the literature focus on mentally demanding tasks. In our case, the higher production rate of the cobot would lead to a higher number of completed assemblies. Hence, we anticipate the task load to be attributed to physical and temporal demand.

### 3.6 Emotion estimation model

For our analysis, we employed a Social Signal Interpretation (SSI) pipeline (Wagner et al., 2013) to capture, process, and estimate emotions from the facial expressions of the participants. To detect the face region, we utilized MediaPipe’s Blaze face detection model (Bazarevsky et al., 2019). Subsequently, we cropped the images within the detected face regions. The cropped face images served as input to a deep-learning model trained to predict emotions. The model classified images into seven discrete emotion classes: Neutral, Happy, Sad, Surprise, Fear, Disgust, and Anger. Additionally, the model provided two continuous values (in the range [−1, 1]) for each image: Valence and Arousal.

The model was trained on the AffectNet dataset (Mollahosseini et al., 2019) following pre-processing as Toisoul et al. (2021), resulting in 218, 827 images split into 7 emotion classes (85% training, 15% validation). Our model consisted of a VGG16 network connected to a fully connected layer, followed by three prediction layers for 7 emotion classes (Softmax activation), valence (Tanh activation), and arousal (Tanh activation). All input images were scaled to 224 × 224 pixels and augmented with techniques such as width shift, height shift, zoom, and horizontal flip. The SGD optimizer with an initial learning rate of 0.001, reduced by a factor of 0.1 after 70,000 steps, was employed. The neural network and training approach was adapted from Prajod et al. (2021, 2022) as it has been validated to learn facial action units, a key aspect of emotion recognition. For discrete emotion classification, the focal loss function (Lin et al., 2017) was used, and for valence and arousal prediction, the shake-shake loss function (Toisoul et al., 2021) was utilized. Early stopping (patience = 5) was employed to prevent over-fitting by halting training when validation loss stagnated for 5 consecutive epochs. As demonstrated by Nunnari et al. (2023), this model achieved performance comparable to the state-of-the-art.

We applied the model to the videos obtained from the three experimental conditions. This yielded a sequence of frame-wise emotion estimations, providing a characterization of each session. To ensure the reliability of the estimations, frames in which no face was detected were excluded from further analysis. Additionally, emotions experienced during the initial period may not be indicative of the participants’ overall experience of the session, as it takes some time for them to fully engage and respond to the robot’s behavior. Therefore, we exclude the estimations from the first 5 min.

### 3.7 HRV features

We extract HRV features from the ECG data collected from the participants. The ECG signals go through a series of cleaning steps before we extract the HRV features. We apply a second-order Butterworth band-pass filter (8–20 Hz) to remove noises in the signal (Elgendi et al., 2010; Prajod and André, 2022). We segment the ECG signals into 1-minute-long segments using a sliding window with shifts of 1 s. We detect the heartbeats using the method proposed by Elgendi et al. (2010). We discard an ECG segment if there is a missing beat or false detections (too many beats). A segment is deemed to have missed a beat if the time between any two consecutive beats is more than 1200 milliseconds (leading to a heart rate < 50 beats per minute). Similarly, if any two consecutive beats occur less than 333.33 milliseconds apart (heart rate > 180 beats per minute), then we consider the segment to have points that are wrongly detected as beats. Using NeuroKit2 (Makowski et al., 2021) python library, we compute 22 HRV features from the time domain, frequency domain, and poincaré plots (Prajod and André, 2022; Heimerl et al., 2023).

Similar to emotion estimation (see Section 3.6), we exclude the data from the initial 5 min of each session. Additionally, we also exclude nine participants who do not have at least 5 min of clean ECG data in each of the sessions.

## 4 Analysis and results

### 4.1 Task load analysis

We first analyze the responses of the participants to the NASA-TLX questionnaire. The mean response values for each condition are reported in Table 1.

**TABLE 1:** The average responses to the NASA-TLX questionnaires (on a 20-point scale) after each condition.

Category	Slow	Fast	Adaptive
Mental demand	4.81	**6.35**	4.95
Physical demand	4.59	**6.84**	5.05
Temporal demand	4.73	**10.54**	6.08
Effort	5.16	**7.76**	5.65
Performance	**7.30**	6.81	6.81
Frustration	**5.35**	**5.35**	4.27

The bold values indicate the highest average score between the three conditions for each category.

The Fast condition resulted in the highest Effort, Mental, Physical, and Temporal demands. The Slow condition scored lowest in these categories. As mentioned in Section 3.5, such a difference was expected in Temporal and Physical demands, but it is interesting to see that the cobot’s production rate affected other categories of task load. However, from the magnitude of the differences, the primary source of task load is the Temporal demand.

Although the number of assemblies was the highest in the Fast condition and lowest in the Slow condition, the perceived Performance was highest for the Slow condition. Another notable observation is that the participants experienced lower frustration in the Adaptive condition.

### 4.2 Emotion estimation analysis

Our emotion analysis primarily focused on the valence and arousal values, as continuous values provide a more dynamic estimation of emotions. To examine the overall emotional estimations per session, we computed the mean valence and arousal values for each participant. Averaging over all participants, the mean valence levels (Slow: −0.025, Fast: −0.018, Adaptive: −0.023) are lowest for the Slow condition and highest for the Fast condition. The mean arousal values (Slow: 0.053, Fast: 0.074, Adaptive: 0.071) also follow a similar trend.

To evaluate the statistical significance of the differences in mean valence and arousal values across the experimental conditions, we conducted a repeated measures ANOVA. The assumptions of homogeneity of variance (Levene test) and normality (Shapiro-Wilk test) were met by the samples, validating the application of the test. There was a statistically significant difference in mean arousal between at least two conditions (F = 8.23, *p* < 0.001). However, there was no significant difference in mean valence between conditions.

To identify specific conditions that exhibited significant variations in mean arousal, we performed *post hoc* pairwise t-tests between every pair of conditions. To account for multiple comparisons, we utilized the Holm correction method for the *post hoc* tests. Post hoc tests indicated that the Slow condition differed significantly in arousal from both the Fast (*p* = 0.012) and the Adaptive (*p* = 0.015) conditions. We found no evidence that there is a significant difference in mean arousal levels between the Fast and the Adaptive conditions (*p* = 0.884).

Although we found a significant difference in arousal levels between Slow and higher-challenge conditions, the mean arousal values in all three conditions are in the range [0, 0.1]. These values are typically associated with a neutral emotional state. So, the facial expressions of the participants are not a good indicator of the perceived challenge level (RQ 1a).

### 4.3 Heart rate variability analysis

One of the commonly used ECG features in Flow detection is the heart rate (HR) (Rissler et al., 2020), which is measured in terms of beats per minute. In this section, we use HR as an example to describe the analysis process. The same analysis is repeated for all 22 HRV features.

**FIGURE 5:**
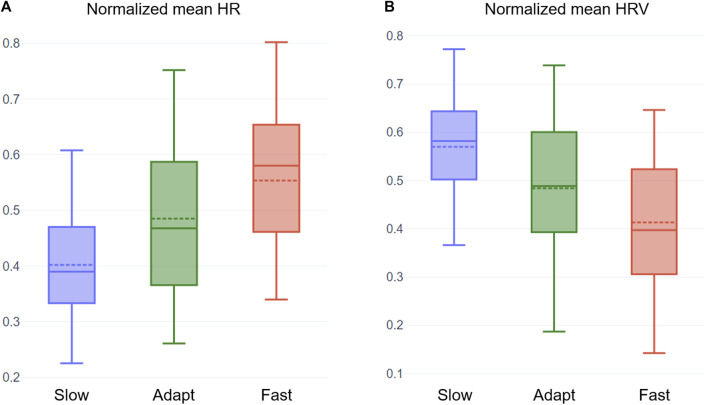
Box plots of the normalized mean HR **(A)** and normalized mean HRV **(B)** of participants in the three conditions. The dotted line on each box plot represents the mean value of the distribution.

To mitigate the effects of individual differences, we applied MinMax normalization to HR and other HRV features of every participant. We plotted the normalized mean HR in the three conditions as visualized in Figure 5A. Similar to emotion estimation, the HR tends to be highest in the Fast condition (mean = 0.554), followed by the Adaptive condition (mean = 0.485), and the lowest in the Slow condition (mean = 0.402).

Similar to Section 4.2, we performed a repeated measure ANOVA on the average normalized HR values. We confirmed that the samples met the assumptions of homogeneity of variance and normality. We obtained a statistically significant difference between at least two conditions (F = 10.59, *p* < 0.01). Post hoc pairwise t-tests with Holm correction revealed a significant difference between the Slow condition and the other two conditions (Fast *p* < 0.001, Adaptive *p* = 0.038). There was only a trend-level difference between average heart rates of Fast and Adaptive conditions (*p* = 0.056).

**FIGURE 6:**
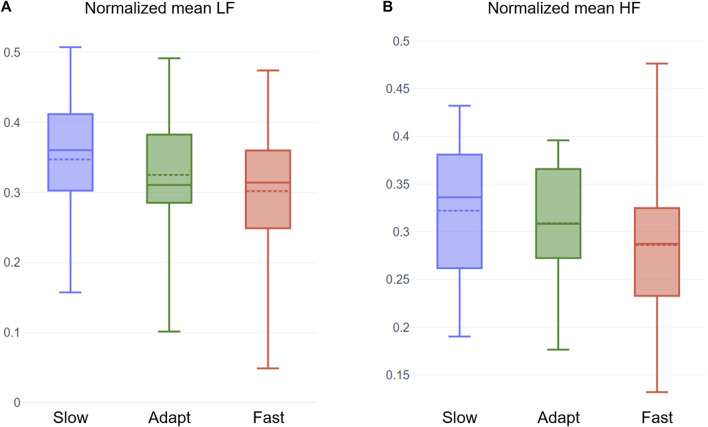
Box plots of the normalized mean LF **(A)** and normalized mean HF **(B)** of participants in the three conditions. The dotted line on each box plot represents the mean value of the distribution.

Our mean HR and HRV analysis show trends similar to the observations of studies in the literature (see Section 2), which predominantly focused on mentally demanding tasks. As seen in Figure 5, HR increases with challenge level and HRV decreases with challenge level. The Adaptive condition resulted in a relatively moderate HR and HRV, which is expected in a challenge-skill balanced condition. We also plotted average LF and HF features as they are often studied in the Flow literature. Both LF and HF show a decreasing trend (see Figure 6), with the highest values in the Slow condition and the lowest in the Fast condition.

Table 2 lists the outcomes of the statistical analysis for all the HRV features that we extracted. The first 13 features are temporal features, the next 5 belong to the frequency domain, and the last 4 are derived from the poincaré plots. Detailed descriptions of these features can be found in Prajod and André (2022). The “*” symbol indicates that the result is deemed statistically significant (p < 0.05). Some of the HRV features, particularly from the temporal domain, show significant differences in the ANOVA test even after *post hoc* Holm *p*-value correction. Hence, HRV features could be good indicators of perceived challenge levels during human-robot collaboration tasks as well (RQ 1b).

**TABLE 2:** Significance test results for the HRV features.

Feature	ANOVA	Post hoc	C1 vs. C2	C1 vs. C3	C2 vs. C3
Time domain features					
HR	p < 0.001 *	*p* = 0.002 *	*p* < 0.001 *	*p* = 0.038 *	*p* = 0.056
Mean NN	p < 0.001 *	*p* = 0.002 *	*p* < 0.001 *	*p* = 0.038 *	*p* = 0.054
SD NN	*p* = 0.017 *	*p* = 0.231			
CV NN	*p* = 0.553	*p* = 0.553			
Median NN	p < 0.001 *	*p* < 0.001 *	*p* < 0.001 *	*p* = 0.018 *	*p* = 0.027 *
Mad NN	p < 0.001 *	*p* = 0.018 *	*p* < 0.001 *	*p* = 0.046 *	*p* = 0.052
RMSSD	*p* = 0.123	*p* = 0.862			
SDSD	*p* = 0.121	*p* > 0.99			
IQR NN	*p* = 0.005 *	*p* = 0.082			
pNN50	*p* = 0.026 *	*p* = 0.255			
pNN20	p < 0.001 *	*p* = 0.005 *	*p* < 0.001 *	*p* = 0.146	*p* = 0.012 *
TI NN	*p* = 0.552	*p* > 0.99			
TI	p < 0.001 *	*p* = 0.002 *	*p* < 0.001 *	*p* = 0.002 *	*p* = 0.595
Frequency domain features					
LF	*p* = 0.017 *	*p* = 0.216			
HF	*p* = 0.020 *	*p* = 0.221			
LF/HF	*p* = 0.334	*p* > 0.99			
LF/Total	*p* = 0.411	*p* > 0.99			
HF/Total	*p* = 0.262	*p* > 0.99			
Poincaré plot features					
SD1	*p* = 0.121	*p* = 0.968			
SD2	*p* = 0.012 *	*p* = 0.183			
SD1/SD2	*p* = 0.285	p > 0.99			
S (Area)	*p* = 0.018 *	*p* = 0.217			

### 4.4 Challenge prediction

The significance test results of heart rate variability features are promising. Hence, using HRV features, we trained simple feed-forward neural networks to predict the challenge level experienced by the participants. We consider two cases:

• two-class prediction—discerning between three conditions (Slow vs. Fast vs. Adaptive)
• three-class prediction—discerning between two conditions (Slow vs. Fast/Adaptive)

In the second case, we combine the data from the Fast and Adaptive conditions. This case was considered after the analyses from previous sections (see Sections 4.2, 4.3), which revealed that, in the majority of cases, there were no significant differences between the Fast and Adaptive (C2 vs. C3) conditions.

We use a neural network architecture comprising an Input layer, and two hidden layers (ReLU activation) with 12 and 6 nodes, respectively. To mitigate over-fitting, we added a Dropout layer (rate = 10%) after the Input layer. Depending on the prediction case, the Output layer (Softmax activation) had 2 or 3 nodes. The models were trained in batches of 128 samples and using an SGD optimizer with a learning rate of 0.01. For robust results on unseen data, we used the leave-one-subject-out (LOSO) evaluation method. The average performance results of the challenge prediction models are reported in Table 3.

**TABLE 3:** LOSO evaluation results of two-class and three-class perceived challenge prediction models.

Model	Accuracy	F1-score
Baseline Three-class (always predicting one class)	0.333	0.167
Our Three-class (low vs. med vs. high challenge)	0.493	0.459
Baseline Two-class (always predicting majority class)	0.667	0.533
Our Two-class (low vs. med + high challenge)	0.707	0.661

The two-class perceived challenge prediction achieves much better performance than the three-class prediction. The heart rate variability analysis in Section 4.3 indicated that this was a plausible outcome. Although the accuracy of the two-class model may not be notably high for a binary classifier, the model performs comparably to the flow detection models in the literature (see Section 2.3). In other words, this performance is comparable to mentally demanding scenarios, indicating that the low accuracy of the model is not due to the physical or temporal demands of the task. Hence, we infer that differentiating between low- and high-challenge conditions using HRV features in a human-robot collaboration scenario is a feasible goal (RQ 2).

In a previous study (Beyrodt et al., 2023), it was observed that when the cobot was faster than the participant, they tended to reappraise the situation and over time start working at their own pace. This could be a plausible reason for the lack of significant difference between the Fast and Adaptive conditions in our study. On the contrary, when the cobot operated at a slower pace, participants were prone to distraction and, consequently, assembling mistakes. Hence, in an industrial setting, it is more crucial to detect low-challenge situations and adapt the cobot’s behavior accordingly. Our two-class perceived challenge prediction model is a promising step toward achieving this objective.

## 5 Discussion

We designed this experiment to study how people respond to different challenge levels in a human-robot collaboration scenario. As seen in Section 4.1, the participants’ responses to the NASA-TLX questionnaire indicate that they experienced different task loads in the three study conditions. Interestingly, the Adaptive condition was perceived as less frustrating than the Slow and Fast conditions. This could be because the Adaptive condition leads to synchronized joint activity, whereas, both Slow and Fast conditions result in one of the collaborating partners waiting for the other.

We anticipated that the Slow and Fast conditions would evoke negative emotions, while the Adaptive condition would facilitate positive emotions conducive to the flow state. Contrary to our expectations, we did not observe significant differences in valence levels among the three conditions. The average valence and arousal levels across all conditions were close to 0, i.e., valence-arousal values close to the neutral state. This suggests that the programmed behavior of the collaborative robot, which closely resembles typical industry scenarios, did not elicit strong negative emotions from the participants.

Our heart rate variability analysis indicates a notable distinction between the Slow condition and the other two conditions (Fast and Adaptive). The Slow condition corresponds to a low-challenge scenario, while the Fast and Adaptive conditions entail higher levels of challenge. Our observations are in line with the results of Keller et al. (2011), where they found a significant difference in mean HRV during the low challenge (Boredom) and higher challenge (Fit, Anxiety) conditions. Similar to our analysis, they found the difference between Fit and Anxiety conditions to be a trend but not significant (*p* < 0.1). Similarly, our plots of LF and HF align with the findings of Tozman et al. (2015) that investigated the physiological response of participants during three simulated driving conditions - Boring, Adaptive, and Anxious. The HF components of HRV are typically associated with parasympathetic activity (relaxation), and hence the observed trend is reasonable. However, while some studies associate LF with sympathetic activation (arousal), others interpret it as a measure of both sympathetic and parasympathetic activities (Electrophysiology, 1996). Using the latter interpretation, we can say that the decreasing trend in LF is a reflection of reduced relaxation with the increase in challenge. This shows that the Adaptive condition led to relatively moderate relaxation and moderate arousal.

Using the HRV features computed from the ECG data of the participants, we trained simple neural networks to predict the three challenge levels. Based on our analysis, we also consider the low (Slow) vs. high (Fast, Adaptive) challenge classification. This two-class prediction model yields much better performance than the three-class model and is on par with the state-of-the-art Flow detection (low vs. high Flow) models (Müller and Fritz, 2015; Rissler et al., 2020; Di Lascio et al., 2021).

In their study, Tozman et al. found that LF and HF components of HRV can distinguish between the three challenge levels. However, our models could not differentiate between the higher task-demand conditions. One possible explanation for this disparity is the difference in the methods used to induce challenge. In their study, participants experienced social evaluation as a stressor in the Anxious condition, which likely contributed to heightened physiological responses. In contrast, our study manipulated the challenge level solely through the robot’s behavior, without incorporating external sources of challenge.

The concept of Flow and the corresponding physiological responses are typically studied using cognitive tasks such as quizzes, puzzles, reading comprehension, etc. Our study shows that robot behavior in human-robot collaboration tasks can influence the level of challenge experienced by the participants, which leads to differences in their physiological responses. We also showed that physiological signals can be used to train models that detect the level of challenge. Such models can be leveraged to adapt the robot’s behavior to match the skill of the operator in order to support Flow in industrial settings.

## 6 Conclusion

From the literature, we recognize the importance of the Flow experience in optimizing worker performance and wellbeing. Hence, we aim to bridge the gap in the study of Flow within industrial settings, an area that has remained relatively unexplored. Moreover, the typical tasks explored in the Flow experience studies are often mentally demanding and diverge significantly from the repetitive and standardized nature of industrial assembly tasks.

To study the emotional and physiological responses in an industrial task, we designed an assembly scenario where an operator and a cobot collaboratively assembled gearboxes. We incorporated three distinct levels of challenge by modifying the cobot’s production rate: low challenge (Boredom), adaptive challenge (Flow), and high challenge (Anxiety). We collected video, ECG, and NASA-TLX questionnaire data from 37 participants. As we anticipated, the primary source of task load stemmed from temporal demand, as opposed to mental demand.

Our investigation focused on how facial emotion estimation (valence and arousal) and HRV features varied across the three challenge conditions. Notably, we observed a significant distinction in mean arousal and five temporal HRV features between the low challenge condition and the other conditions. Additionally, we identified a noteworthy trend-level difference between the adaptive and high-challenge conditions. Importantly, despite the shift in the nature of the task load, our findings align with existing literature.

Building on our analysis, we proceeded to develop HRV-based models capable of predicting perceived challenge levels. The implementation of such models carries the potential to facilitate dynamic adjustments in challenge levels based on real-time feedback, thereby fostering the Flow experience among cobot workers. Our research contributes to the human-centered paradigm of Industry 5.0 and lays the foundation for adaptive work environments geared towards enhancing worker experiences and wellbeing.

Looking ahead, it would be valuable to explore emotional and physiological responses over extended timeframes, as we anticipate that more pronounced distinctions may emerge. Additionally, it would be interesting to delve into the operator’s experience when interacting with a comprehensive system that dynamically adjusts challenge levels in real time. This line of investigation could shed light on the applicability and effectiveness of adaptive challenge modulation. Moreover, while our current study successfully recreated an industrial assembly task within a controlled laboratory setting, a natural progression would be to evaluate worker experiences in actual industrial environments. Such research holds promise in shaping future workplace practices and promoting optimal worker experiences.

## Data Availability

The datasets presented in this article are not readily available due to concerns regarding privacy and participant anonymity. Requests to access the datasets should be directed to the corresponding author.
